# Flow-induced Reorganization of Laminin-integrin Networks Within the Endothelial Basement Membrane Uncovered by Proteomics

**DOI:** 10.1074/mcp.RA120.001964

**Published:** 2020-04-24

**Authors:** Eelke P. Béguin, Esmée F. J. Janssen, Mark Hoogenboezem, Alexander B. Meijer, Arie J. Hoogendijk, Maartje van den Biggelaar

**Affiliations:** 1 Department of Molecular and Cellular Hemostasis, Sanquin Research, Amsterdam, The Netherlands; 2 Department of Biomolecular Mass Spectrometry, Utrecht Institute for Pharmaceutical Sciences (UIPS), Utrecht University, Utrecht, The Netherlands

**Keywords:** Extracellular matrix, inflammation, label-free quantification, mass spectrometry, SILAC, basement membrane, endothelium, flow, integrin, laminin

## Abstract

The vessel wall is continuously exposed to hemodynamic forces generated by blood flow. Endothelial mechanosensors perceive and translate mechanical signals via cellular signaling pathways into biological processes that control endothelial development, phenotype and function. To assess the hemodynamic effects on the endothelium on a system-wide level, we applied a quantitative mass spectrometry approach combined with cell surface chemical footprinting. SILAC-labeled endothelial cells were subjected to flow-induced shear stress for 0, 24 or 48 h, followed by chemical labeling of surface proteins using a non-membrane permeable biotin label, and analysis of the whole proteome and the cell surface proteome by LC-MS/MS analysis. These studies revealed that of the >5000 quantified proteins 104 were altered, which were highly enriched for extracellular matrix proteins and proteins involved in cell-matrix adhesion. Cell surface proteomics indicated that LAMA4 was proteolytically processed upon flow-exposure, which corresponded to the decreased LAMA4 mass observed on immunoblot. Immunofluorescence microscopy studies highlighted that the endothelial basement membrane was drastically remodeled upon flow exposure. We observed a network-like pattern of LAMA4 and LAMA5, which corresponded to the localization of laminin-adhesion molecules ITGA6 and ITGB4. Furthermore, the adaptation to flow-exposure did not affect the inflammatory response to tumor necrosis factor α, indicating that inflammation and flow trigger fundamentally distinct endothelial signaling pathways with limited reciprocity and synergy. Taken together, this study uncovers the blood flow-induced remodeling of the basement membrane and stresses the importance of the subendothelial basement membrane in vascular homeostasis.

## Abstract (precis)

Hemodynamics is crucial for the function of vascular endothelial cells, and its disturbance has been linked to vascular diseases. Here, we combined quantitative mass spectrometry and immunofluorescence studies to dissect the endothelial response to flow-exposure. Our data uncover extensive flow-induced remodeling of the laminin network and unveil the relocation of laminin-associated integrins on the basal endothelial surface. These data highlight the importance of the endothelial basement membrane in vascular homeostasis and emphasize its plasticity.

## Graphical Abstract

**Figure:**
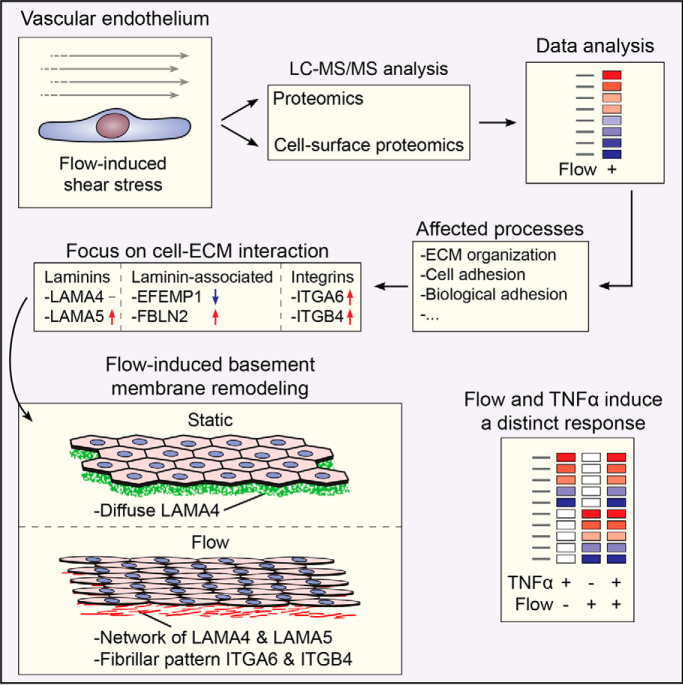

**Highlights**

- Proteomics uncovers the flow-induced remodeling of the endothelial basement membrane.
- Flow alters the composition and localization of the laminin-integrin network.
- Flow induces proteolytic processing of LAMA4, resulting in shedding of LG4–5 region.
- TNFα- and flow-exposure induce a distinct proteomic signature with limited interplay.

The vascular network is crucial to supply tissues with oxygen and nutrients and to remove waste products. The inner lining of this network is formed by a monolayer of endothelial cells (ECs), which provide a dynamic interface between blood and underlying tissues and actively mediate various physiological processes, including regulation of the vasomotor tone, vessel wall permeability and inflammation (1). The EC phenotype and function are modulated by hemodynamic forces in the circulation, including blood flow-induced shear stress. Depending on the local flow type and its strength, the morphology of ECs ranges from polygonal, in which the cells are randomly orientated, to spindle-like, in which cells are aligned in the direction of the flow. Aligned cells are characterized by enhanced cell survival, an effective barrier function, and limited capacity to support leukocyte transmigration. In contrast, the non-aligned phenotype is associated with enhanced cellular turnover, leakiness to plasma macromolecules, and increased leukocyte adhesion (2, 3). It has therefore been proposed that absence of flow elicits a pro-inflammatory effect (4). However, the molecular mechanisms that underlie the endothelial hemodynamic response are incompletely understood as well as the effects of hemodynamics on the EC's interaction with its environment. In addition, multiple studies have demonstrated a link between flow and inflammation (5–10), yet the associated proteomic alterations remain to be explored.

Functional studies have established that ECs respond to changes in hemodynamics through a set of mechanosensitive receptors, which include PECAM1 (platelet and endothelial cell adhesion molecule 1), VEGFR2 (vascular endothelial growth factor receptor 2, KDR), VE-Cadherin (vascular endothelium cadherin, CDH5) (11, 12), PAR1 (proteinase-activated receptor 1, F2R) (13), ion channels (14) and integrins (15, 16). Sensing of flow by the endothelial mechanosome initiates signaling cascades that activate a variety of transcription factors, including KLF2 (kruppel-like factor 2) (17) and KLF4 (18), and inhibit others, such as YAP/TAZ (Yes-associated protein, tafazzin) (19) and proteins of the NF-κB (nuclear factor kappa-light-chain-enhancer of activated B cells) complex (20). These signaling events eventually lead to a transient increase in endothelial barrier function (21), an increased production of the vasodilatory agent nitric oxide via up-regulation of NOS3 (nitric oxide synthase 3, eNOS) and a transient inhibition of cell proliferation to prioritize flow adaptation (22). In addition, flow-induced mechanosensory signaling initiates remodeling of the actin cytoskeleton and subendothelial extracellular matrix (ECM), resulting in alterations in cell anchorage to the underlying sheet-like specialized ECM: the basement membrane (BM) (23, 24). However, the details of this altered cell-ECM interaction remain to be elucidated. Recently, a systems biology analysis of the longitudinal response of ECs to shear stress at a transcriptional level exposed the dynamics of several functional pathways, including cell cycle, oxidative stress, and inflammation (25). However, how these transcriptomic changes translate into molecular mechanisms that guide the flow-induced phenotypical and functional transition, specifically at the cell surface and extracellular region, has remained unclear.

To address this issue, we performed a global quantitative proteomic analysis on the effects of flow on ECs and particularly focused on cell surface proteins by employing a quantitative chemical footprinting approach. Combined, our data show that flow most prominently induced changes in proteins involved in cell adhesion and ECM organization, most notably in the laminin and integrin protein families. By employing immunofluorescence microscopy, we subsequently uncovered a drastic remodeling of the ECM and cellular adhesion molecules, including laminin α4 (LAMA4), α5 (LAMA5), fibulin-2 (FBLN2), EGF Containing Fibulin Extracellular Matrix Protein 1 (EFEMP1 or fibulin-3), integrin α6 (ITGA6) and integrin β4 (ITGB4). Furthermore, colocalization of integrin α6β4, LAMA4 and LAMA5 suggests that integrin α6β4 may provide a cellular link to the laminin ECM to withstand flow-induced mechanical forces. In addition, we showed that the response of ECs to tumor necrosis factor α (TNFα) was independent on flow-adaptation, indicating that flow and inflammation trigger distinct signaling pathways. Combined, these data provide molecular details on the flow-induced remodeling of the endothelial BM and expose a role for integrin α6β4 in cell-matrix adhesion in flow-conditions.

## EXPERIMENTAL PROCEDURES

### 

#### 

##### Primary Cell Culture

Blood Outgrowth Endothelial Cells (BOECs) (26) were obtained from blood from healthy volunteers in accordance with Dutch regulations and the Declaration of Helsinki after approval from the Sanquin Ethical Advisory Board. Written informed consent was given by all participants. BOECs originating from 3 healthy volunteers (mixed sexes) were pooled and used in the described experiments. Culture flasks and dishes were coated with 50 μg/ml collagen type I (BD biosciences) before use. BOECs were grown in Endothelial Cell Growth Medium (Promocell, Heidelberg, Germany) supplemented with 18% fetal bovine serum (FBS) (Bodinco, Alkmaar, The Netherlands) and maintained in a humidified 5% CO_2_, 37 °C incubator. For SILAC labeling, BOECs were maintained for 5 passages as previously described (27) using custom-made EBM-2 medium (Lonza) supplemented with Endothelial SingleQuots, and containing 18% 1 kDa dialyzed FBS (Bodinco) and isotope labeled amino acids (light: Arg0 & Lys0, medium: Arg6 & Lys4, heavy: Arg10 & Lys8, Cambridge Isotopes). Primary HUVEC (Lonza) were maintained in Endothelial Cell Growth Medium (Promocell) containing 2% FBS, lung hMVEC (Lonza) in Enhanced Endothelial Cell Growth Medium (PeloBiotech) containing 5% FBS.

##### Flow-exposure of Endothelial Cells

BOECs were seeded in a ring-shaped culture dish, formed by placing a Ø 54 mm (outer diameter) Petri dish bottom in the middle of a Ø 86 mm (inner diameter) Petri dish. In the middle of the inner dish a magnet was placed, underneath the outer dish a T-shaped metal strip. The formed ring-shaped dish was then coated with 50 μg/ml collagen type I (BD biosciences), seeded with BOECs in 20 ml medium and incubated overnight. The next day these Petri dishes were placed on a Stuart SSL3 gyrorocker platform on top of an 11 cm high elevated platform, which was set to rotate at 60 RPM and placed in a humidified 5% CO_2_ 37 °C incubator. Within all experiments, the endpoints of the different experimental conditions were synchronized, *i.e.* the “static” samples were maintained for 48 h in static conditions before lysis/fixation.

##### Quantitative MS Analysis of Flow-treated SILAC-labeled ECs

SILAC-labeled BOECs were seeded in the ring-shaped culture dishes as described above and subjected to flow when appropriate. Mass spectrometry sample preparation and acquisition, as well as cell surface labeling were performed as previously described (27). Briefly, SILAC labeled BOECs were washed and incubated for 30 min at 4 °C with 3 mm EZ-Link sulfo-NHS-LC-biotin (ThermoFisher Scientific). Excess label was quenched with 100 mm Glycine, and cells were lysed in 4% SDS, 100 mm DTT, 100 mm Tris, pH 7.5, supplemented with HALT phosphatase and protease inhibitor mixture (Thermo Scientific). Proteins were proteolytically digested with trypsin (Promega, cleaves after Lys and Arg) or chymotrypsin (Thermo Scientific, cleaves after Tyr, Trp, and Phe) using filter-aided sample preparation (FASP) (28). Biotin-labeled peptides were pulled-down using a SigmaScreen Streptavidin high capacity coated plate (Sigma Aldrich). For compatibility with downstream LC/MS-MS analysis, in contrast to commonly used enzymatic release or reductive elution, biotin-labeled peptides were eluted with 70% acetonitrile, 5% formic acid as described previously (27) and subsequently subjected to C18 StageTip (29) desalting and mass spectrometry analysis. For proteome samples 50 μg tryptic digests were fractionated using a Strong Anion eXchange fractionation using Empore Anion and Cation Exchange-SR Extraction Disks (3m) (30), and peptides were desalted using C18 (3 m) StageTips (30). Peptides were separated by nanoscale C18 reverse chromatography coupled online to an Orbitrap Fusion Tribrid mass spectrometer (Thermo Scientific) via a nanoelectrospray ion source at 2.15 kV.

##### Label-free Quantitative MS Analysis of Flow/TNFα Treated ECs

After 48 h of flow-exposure, 10 ng/ml TNFα was added to the medium when appropriate and flow/static incubation was continued for 24 h. Cells were prepared for MS analysis as described (31) with minor modifications. Briefly, ECs were lysed in 1% sodium deoxycholate 10 mm TCEP, 40 mm chloroacetamide, 100 mm Tris-HCl pH 8.0 supplemented with 1x HALT protease/phosphatase inhibitor (Thermo Scientific). Lysates were incubated for 5 min at 95 °C and sonicated for 10 min in a sonifier bath (Branson model 2510), after which trypsin (Promega) was added in a 1:50 (w/w) protein ratio. Peptides were loaded on in-house prepared SDB-RPS (Empore) StageTips, and eluted into 3 fractions subsequently using: buffer 1 (100 mm ammonium formate, 40% (v/v) acetonitrile, 0.5% (v/v) formic acid), buffer 2 (150 mm ammonium formate, 60% (v/v) acetonitrile, 0.5% (v/v) formic acid) and buffer 3 (5% (v/v) ammonium hydroxide, 80% (v/v) acetonitrile). Samples were vacuum dried and peptides were dissolved in 2% (v/v) acetonitrile, 0.1% (v/v) TFA, and subjected to MS-analysis. Peptides were separated by nanoscale C18 reverse chromatography coupled on line to an Orbitrap Fusion Lumos Tribrid mass spectrometer (Thermo Scientific) via a nanoelectrospray ion source at 2.15 kV. Buffer A was composed of 0.5% acetic acid and buffer B of 0.5% acetic acid, 80% acetonitrile. Peptides were loaded for 17 min at 300 nl/min at 5% buffer B, equilibrated for 5 min at 5% buffer B (17–22 min) and eluted by increasing buffer B from 5–27.5% (22–122 min) and 27.5–40% (122–132 min), followed by a 5 min wash to 95% and a 6 min regeneration to 5%. Survey scans of peptide precursors from 375 to 1500 *m*/*z* were performed at 120K resolution (at 200 *m*/*z*) with a 4 × 10^5^ ion count target. Tandem mass spectrometry was performed by isolation with the quadrupole with isolation window 0.7, HCD fragmentation with normalized collision energy of 30, and rapid scan mass spectrometry analysis in the ion trap. The MS2 ion count target was set to 3 × 10^4^ and the max injection time was 20 ms. Only those precursors with charge state 2–7 were sampled for MS^2^. The dynamic exclusion duration was set to 30 s with a 10 ppm tolerance around the selected precursor and its isotopes. Monoisotopic precursor selection was turned on. The instrument was run in top speed mode with 3 s cycles. All data were acquired with Xcalibur software.

##### MS Data Analysis and Visualization

Raw files were processed in the Maxquant 1.5.3.30 computational platform (32) to identify proteins and peptides by querying the uniprot database for human proteins (release 3–2017, 70947 entries) using the andromeda search engine. Default settings of Maxquant included the following parameters: (1) precursor mass tolerance of 4.5 ppm, (2) fragment mass tolerance of 20 ppm, (3) fixed modification: cysteine carbamidomethylation, (4) variable modifications: methionine oxidation and N-terminal acetylation, (5) an FDR of 1% for proteins and peptides, (6) a minimum score of 0 for unmodified and 40 for modified peptides, and (7) a maximum of two missed cleavages.

For the TNFα-flow data set, the label-free quantification (LFQ) was enabled with at least 2 ratios per protein. For SILAC samples multiplicity was set to 3, SILAC pairs were entered and the re-quantify option was enabled. The 'match between runs' option was enabled for all samples and protein quantification was based on unique peptides. For the cell surface proteome samples an optional modification for the biotin label (339.16166 Da) was added. Two subsequent streptavidin pull-downs were treated as fractions of 1 experiment. Peptides were generated using trypsin (whole cell proteome and cell surface proteome) or chymotrypsin (cell surface proteome). All assigned sequences, as well as all identified proteins of the SILAC proteome, cell surface proteomes and the label-free quantifications can be found in supplemental Tables S7–S14. Maxquant output tables were opened in Rstudio version 1.1.383 (R version 3.4.2) and reverse, potential contaminants and 'only identified by site' entries were filtered out. No other data points such as outliers were excluded from the subsequent analyses. SILAC ratios were log_2_ transformed and filtered for at least 3 valid values in at least one of the conditions. Selection criteria for class I phosphopeptides, *i.e.* a localization probability of >0.75 and a score difference of >5 (33), were used for biotinylated peptides. Statistical analysis was performed using a linear model without intercept. The distribution of SILAC ratios was inspected and was normally distributed. A Benjamini-Hochberg multiple testing correction was applied to the *p* values. Values were considered significant and relevant if *p* < 0.05 and log_2_ fold change >1 for SILAC ratios and LFQ intensity values.

GO-term enrichment analysis for the SILAC data was performed by comparing all affected proteins to all quantified proteins using the hypergeometric method from goseq 1.24.0 (34), with an FDR adjusted *p* value cutoff of 0.05. Enriched terms were grouped and visualized using gogadget (35) hierarchical clustering of overlap indices (with the ward.D agglomeration method and Euclidean distance). STRINGdb analysis was performed using the stringApp plug-in 1.2.2 (36) for Cytoscape 3.6.1 (37) with a confidence interval cutoff of 0.7. For the LFQ data and the cell surface proteome data GO-term enrichment analysis was performed in Cytoscape 3.4.0 (37) using the BiNGO plug-in (38) and this was visualized in Graphpad Prism 7.04. For the LFQ data this analysis was performed by comparing all affected proteins to all quantified proteins. For the cell surface data biotin-modified proteins were compared with all quantified proteins. Scatter plots of MS data were visualized using Graphpad Prism 7.04.

##### Immunofluorescence Imaging

**Fig. 1.**
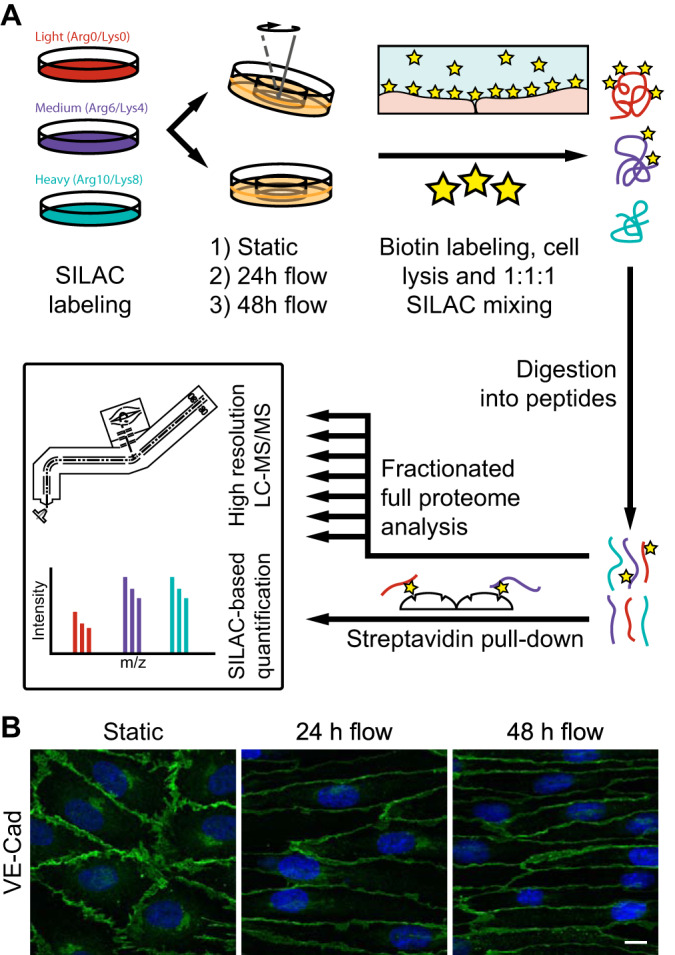
**Mass spectrometry workflow and flow-induced morphological changes in endothelial cells.** *A*, Proteomics workflow. SILAC-labeled BOECs were treated with flow or incubated statically, and labeled with a sulfo-NHS-LC- biotin compound at 4 °C. SILAC-labeled cell lysates were mixed in a 1:1:1 protein ratio, proteins digested with trypsin or chymotrypsin and peptides subjected to streptavidin pull- down. 50 μg of the mixed lysates was used for strong anion exchange (SAX) fractionation. All samples were subjected to high resolution MS. *B*, Immunofluorescence maximum intensity projections of BOECs subjected to flow for 0, 24 or 48 h. Cells were stained for VE-cadherin (green) and with HOECHST (blue). Micrographs are representative for 3 independent experiments. The scale is 10 μm.

For immunofluorescence and immunoblot analyses, BOECs (pool of 3 donors) were grown in Endothelial Cell Growth Medium (Promocell). Tissue culture treated polymer coverslips (Ibidi, #1.5) were mounted in the ring-shaped flow channel before cells were seeded. After flow/static incubation, cells were washed 3x with PBS, and subsequently fixed for 15 min in 4% paraformaldehyde (PFA, Electron Microscopy Sciences) in PBS. Samples were washed 3x time with PBS and PFA was quenched by incubating 15 min in 50 mm NH_4_Cl/PBS. Next, samples were washed with PBS, blocked and permeabilized 15 min in blocking buffer (1% [w/v] BSA [Merck], 0.1% saponin in PBS), and subjected to antibody staining in blocking buffer. Between staining steps samples were washed 3×. After primary and secondary antibody incubation, samples were washed 2x with PBS, incubated 5 min with 1 μg/ml HOECHST 33342 (Life tech) in PBS, washed with PBS and mounted in Aqua poly/mount (Polysciences Inc). Imaging for Fig. 1, 3, 4*C*–4*D*, 5 as well as supplemental Fig. S1, S4, and S5 was performed on a Leica TCS SP8 confocal scanning microscope system, equipped with a 63× Plan-Apochromat 1.40 oil immersion objective. Imaging for Fig. 4*E* and supplemental Figs. S6–S8 was performed on a Zeiss 980 with Airyscan 2, equipped with a 40x Plan-Apochromat 1.30 oil immersion objective. Images were airyscan processed, stitched and maximum intensity projected using the Zen 3.0 software. Further processing was performed using the ImageJ (39) Fiji (40) package. The following antibodies were used: Rat IgG2b α ITGB4 555719 (BD Biosciences); Rat IgG2a α ITGA6 MAB13501 & Sheep polyclonal α LAMA4 AF7340 (R&D systems); Rabbit IgG polyclonal α FBLN2 PA5–51665 (Thermo Scientific); mouse IgG1 α EFEMP1 SC-365224 & Goat IgG polyclonal α VE-cadherin C-19 (Santa Cruz Biotechnology); mouse IgG2a α ITGB1 P4C10 (Millipore); mouse IgG2a α LAMA5 4C7 (Abcam). Secondary: Goat α mouse AF488 A11001, Goat α Rat-AF568 A11077, Donkey α Sheep-AF488 A11015, Donkey α Goat-AF568 A11057, Goat α Rabbit-AF568 A11011, Streptavidin-AF488 S11223 (ThermoFisher Scientific).

##### Immunoblot Analysis

For immunoblot analysis BOECs were subjected to flow as described. Next, BOECs were washed 3× with PBS, lysed in 2x sample buffer (125 mm Tris, 2% SDS, 20% glycerol, 0.02% Bromphenol blue, 20 mm DTT), boiled for 10 min, passed 5× through a 29G needle, spun down 15 min at 16,000 × *g*, and filled up to 1× loading buffer. An estimated 20 μg sample was loaded per well on a NuPAGE 3–8% Tris-Acetate gel (ThermoFisher Scientific). Proteins were separated and transferred to an iBlot nitrocellulose blot (ThermoFisher Scientific). Blot was blocked for 1 h in blocking buffer (4% BSA, 0.1% Tween-20 in TBS (Tris-buffered saline), incubated overnight in Sheep polyclonal αLAMA4 (AF7340, R&D systems) in blocking buffer, washed 3× with TBS 0.1% Tween-20, incubated 1 h in Donkey α Sheep-HRP (713–035-147, Jackson ImmunoResearch) in blocking buffer, and developed using BM chemiluminescence blotting substrate (Roche). The loading control was stained using Rabbit IgG α α-tubulin (Ab52866, Abcam) and Swine α Rabbit-HRP (P0399, DAKO).

##### Analysis of Published Transcriptomics Data

RNA fastq files from (25) were downloaded from GEO (GSE103672). Sequences were aligned to the human hg38 genomic reference sequence using Salmon (41). Differential expression analysis was performed using DESeq2 (42), applying a significance threshold of a Benjamini-Hochberg multiple testing corrected *p* value of <0.05 and log_2_ fold change of >1.

##### Experimental Design and Statistical Rationale

For the SILAC-based MS assessment, a pool of BOECs from 3 healthy donors was split in 3, maintained for 5 passages and subjected to the experimental conditions (*n* = 3). For the label-free quantification-based MS analysis a pool of BOECs from 3 healthy donors was split in 12 and subjected to the experimental conditions (*n* = 3). Statistical analysis was performed using a linear model without intercept. A Benjamini-Hochberg multiple testing correction was applied to the *p* values. All displayed immunofluorescence and immunoblot data are representative images of 3 independent biological replicates employing distinct pools of BOECs from 3 healthy donors.

## RESULTS

### 

#### 

##### Shear Stress Induces Proteomic Alterations that Affect a Range of Cellular Processes, Including Cell-matrix Adhesion

**Fig. 2.**
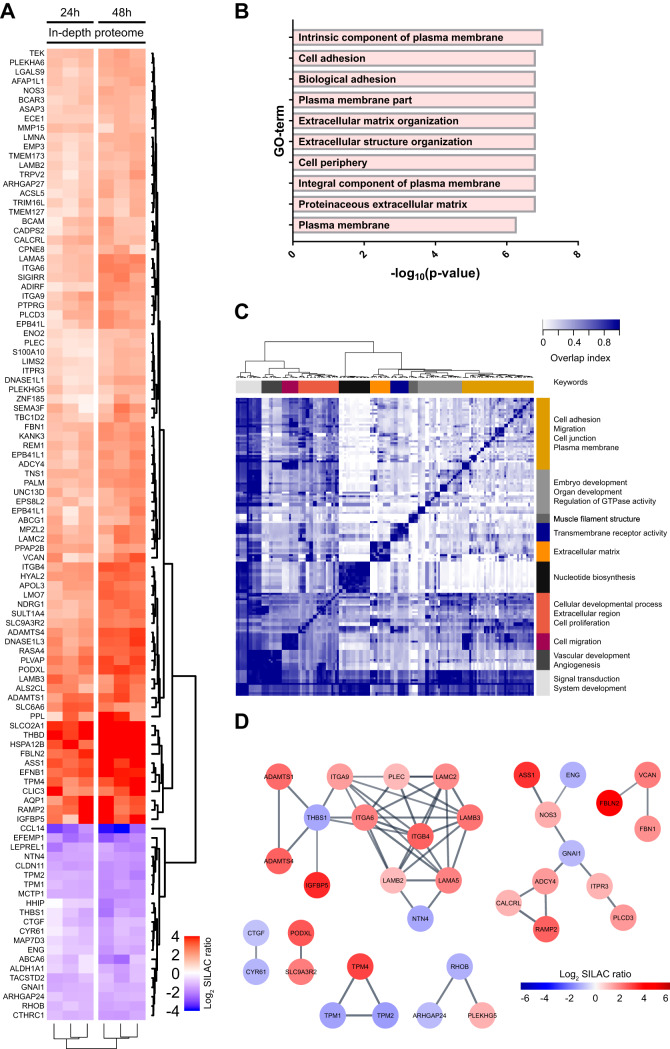
**Flow induces a protein signature characterized by changes in ECM and cell adhesion proteins.** *A*, Heatmap of flow-induced changes in the EC proteome. The colors represent the log_2_ SILAC ratios. Blue represents decreased and red increased values in flow-stimulated samples. Of the 5202 proteins quantified in at least 3 replicates in one of the conditions, 104 changed more than 2-fold upon flow-exposure. A pool of BOECs originating from 3 healthy volunteers was passaged 5× to ensure incorperation of SILAC amine acids. *B*, Overlap-based clustering of enriched GO- terms in flow-affected proteins. Keywords represent the most notable GO-terms in a group. *C*, Top 10 enriched GO-terms. *D*, String analysis of affected proteins. Depicted are only the proteins which have connections in the analysis with a confidence interval cutoff of 0.7. The node colors represent the log2 SILAC ratios.

To assess the effects of flow-induced shear stress, ECs were cultured to confluency in a ring-shaped flow chamber, and subjected to flow for 24 and 48 h using a gyrorocker (Fig. 1*A*). The endpoints of the samples were synchronized to equalize culturing times between samples. Flow exposure resulted in an altered morphology within 24 h, and the ECs were elongated and completely aligned in the direction of the flow after 48 h (Fig. 1*B*). To assess flow-induced differences in protein expression profiles in a quantitative manner, ECs were metabolically labeled using SILAC, and after cell lysis, tryptic digests were subjected to mass spectrometry (MS) analysis (Fig. 1*A*). Using this approach, we quantified 5200 proteins (supplemental Table S1), of which 104 were statistically significantly in- or decreased in flow conditions (Fig 2*A* and supplemental Table S1). Notably, most of these proteins (*n* = 83) were more abundant in the flow-treated cells and only a fraction (*n* = 21) decreased upon shear stress-stimulation (Fig. 2*A*). All affected proteins were consistently in- or decreased in the 24 and 48 h time-points. We complemented whole cell proteomics with the cell surface chemical footprinting approach that we have previously developed to detect intramolecular changes and altered protein-protein interactions (27) (Fig. 1*A*). Therefore, ECs were labeled with a non-membrane permeable biotin label, which was predominantly present at the apical surface of the cells and at the intercellular junctions, as indicated by junctional marker VE-cadherin (supplemental Fig. S1). Using this approach, we quantified a total of 1763 biotin-labeled sites, of which 340 were affected by shear stress (supplemental Table S2–S3, supplemental Fig. S2). In general, the SILAC ratios in the cell surface proteomics data corresponded closely with the SILAC ratios in the whole cell proteomes (supplemental Fig. S2*A*). Like the whole cell proteome, affected proteins in the cell surface proteome were enriched for proteins associated with the plasma membrane, cell adhesion and the extracellular matrix (supplemental Fig. S2*B*).

Flow-induced morphological changes in ECs were accompanied by altered protein levels of various flow-associated proteins, including the nitric oxide-producing enzyme NOS3 (43), HYAL2 (hyaluronidase 2) (44, 45), TEK (TEK Receptor Tyrosine Kinase) (45), THBD (thrombomodulin) (17), ADAMTS1 (a disintegrin and metalloproteinase with thrombospondin motifs 1) (46), and THBS1 (thrombospondin) (47). In addition to these hallmark proteins, we identified regulation of proteins that have not been previously associated with flow-exposure. To gain insight in the functional consequences of these changes, we subsequently conducted a gene ontology (GO)-term enrichment analysis. This analysis yielded 163 GO-terms that were significantly enriched in the group of proteins affected by flow, including ECM-, adhesion-, and cell surface-related terms (Fig. 2*B* and supplemental Table S4). To display the full array of enriched GO-terms, we subsequently clustered these based on their protein overlap (Fig. 2*C*). This analysis indicated that flow affected the abundance of proteins associated with a wide array of processes, ranging from tube development to nucleotide biosynthesis and cell adhesion. In parallel, we performed a STRINGdb analysis to assess the group of flow-affected proteins for functional networks (Fig. 2*D*). This analysis revealed a closely connected network of proteins with various ECM-related functions, including structural ECM proteins (laminins, VCAN [versican]), enzymes (MMP15 [matrix metallopeptidase 15], ADAMTS1&4), and cell adhesion molecules (integrins, BCAM [basal cell adhesion molecule]). In addition, this analysis exposed clusters of proteins related to regulation of the actin cytoskeleton (tropomyosin TPM1, TPM2, TPM4, as well as RHOB [ras homolog family member B], ARHGAP24 [Rho GTPase activating protein 24] and PLEKHG5 [pleckstrin homology and RhoGEF domain containing G5]), nitric oxide production (ASS1 [argininosuccinate synthase 1] and NOS3) and vasodilation (CALCRL [calcitonin receptor like receptor] and RAMP2 [receptor activity modifying protein 2]). Combined, these quantitative mass spectrometry data reveal an important role for cell-matrix adhesion in response to flow.

##### Flow Induces Remodeling of the Laminin Network and Redistribution of Laminin-associated Proteins

**Fig. 3.**
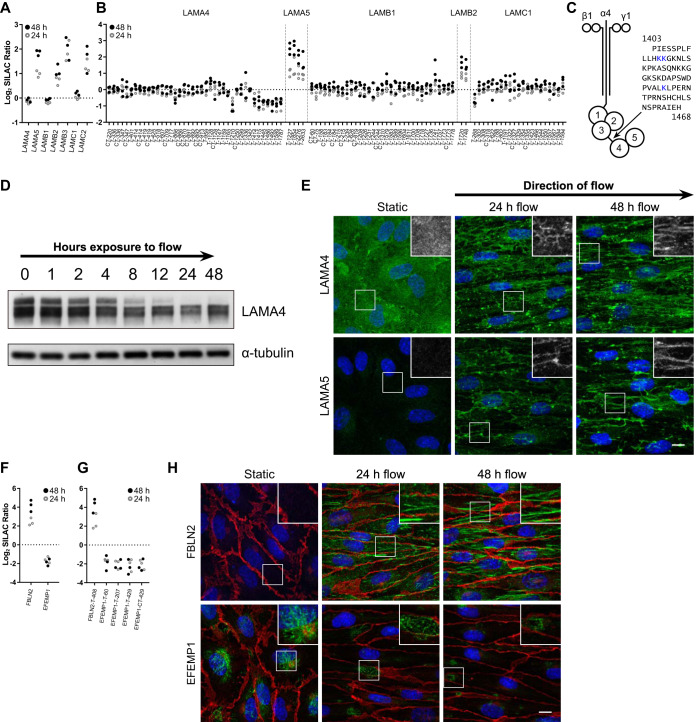
**Flow induces remodeling of the endothelial extracellular matrix.** *A*, Proteome and (*B*) cell surface proteome data of identified laminins. T or CT represents the enzyme used for protein digestion. The depicted numbers represent the biotin-modified lysine residue. *C*, Schematic representation of laminin 411 with the linker region between domains LG3 and LG4. The blue lysine residues were found to be decreased under flow conditions (as depicted in panel *B*). *D*, Immunoblot analysis of LAMA4 in ECs exposed to flow over time. α-tubulin was used as a loading control. *E*, Protein distribution of LAMA5 and LAMA4 after flow-exposure. Green represents LAMA4 or LAMA5, blue is HOECHST. The scale bar is 10 μm. Depicted are maximum intensity projections. *F*, Proteome and (*G*) cell surface proteome data of identified fibulins. *H*, Immunofluorescence analysis of EFEMP1 and FBLN2 abundance and distribution affected by flow. Red is VE-cadherin, blue is HOECHST. Depicted are maximum intensity projections. Micrographs are representative for 3 independent experiments. The scale bar is 10 μm.

One protein family that was considerably affected by flow exposure was the laminin protein family (Fig. 2*D*). Laminins form α/β/γ heterotrimers and are key constituents of the endothelial basement membrane. The BM of ECs contains mainly laminins 411 (α4, β1, γ1) and 511, and in some tissues 421 and 521 (48). Our whole cell proteomics data showed that laminin α5 (LAMA5), β2 (LAMB2), β3 (LAMB3) and γ2 (LAMC2) levels increased, whereas the abundance of laminin α4 (LAMA4), β1 (LAMB1) and γ1 (LAMC1) remained unaffected (Fig. 3*A*). In agreement with these data, cell surface proteomics revealed an increased abundance of LAMA5 and LAMB2 at the cell surface (Fig. 3*B*). This effect was time dependent with more pronounced changes in protein levels after 48 h as compared with 24 h (Fig. 3*A*–3*B*). Interestingly, our cell surface proteomics approach indicated that the abundance of the C terminus of LAMA4 decreased in flow conditions (Fig. 3*B*), whereas the total level of LAMA4 was unaffected (Fig. 3*A*). We hypothesized that this was because of a proteolytic cleavage resulting in the shedding of the LG4–5 region (Fig. 3*C*), which reduces the molecular weight of the residual LAMA4 by about 43–45 kDa (49). In line with this hypothesis, immunoblot analysis showed 2 forms of LAMA4 with a difference in molecular weight corresponding to the LAMA4 C-terminal fragment, of which the heavier band disappeared under flow exposure (Fig. 3*D*).

To gain further insight into flow-induced alterations in the laminin network, we next assessed the cellular localization of LAMA4 and LAMA5 in static and flow conditions. In static conditions, LAMA4 showed a diffuse staining pattern, whereas little LAMA5 staining was detected (Fig. 3*E*). In flow conditions, a crude network-like pattern was observed for both LAMA4 and LAMA5 (Fig. 3*E*), although a high degree of heterogeneity was present throughout the samples. Remarkably, specifically after 48 h, both LAMA4 and LAMA5 were localized in long streaks that were present in the direction of the flow. Subsequent analysis of the relative localization of LAMA4 and LAMA5 indicated that these proteins displayed a high degree of co-localization, specifically after 48 h (supplemental Fig. S4).

Laminins not only play a key role in the assembly of the BM (50, 51), but are also involved in the determination of its molecular composition. In line with this concept, we observed that the laminin-associated proteins FBLN2 and EFEMP1 were among the most differentially abundant proteins in our mass spectrometry-based analysis (Fig. 3*F*–3*G*). The MS data indicated that the abundance of FBLN2 (fibulin-2) was increased upon exposure to flow, whereas the abundance of EFEMP1 (fibulin-3) was decreased. In agreement with these data, immunofluorescence analysis showed limited staining of FBLN2 in static conditions, and a bright staining upon exposure to flow, which appeared in a fibrillar pattern aligning in the direction of the flow (Fig. 3*H*). In contrast to FBLN2, we observed cell-associated networks of EFEMP1 (fibulin-3) in static conditions, which mostly disappeared in flow conditions (Fig. 3*H*). Combined, these data indicate that flow exposure results in a drastic reorganization of the structure and composition of the endothelial BM.

##### Shear Stress Induces a Cell-aligned Fibrillar Distribution of Integrin α6β4 Which Co-localizes with the Laminin Network

**Fig. 4.**
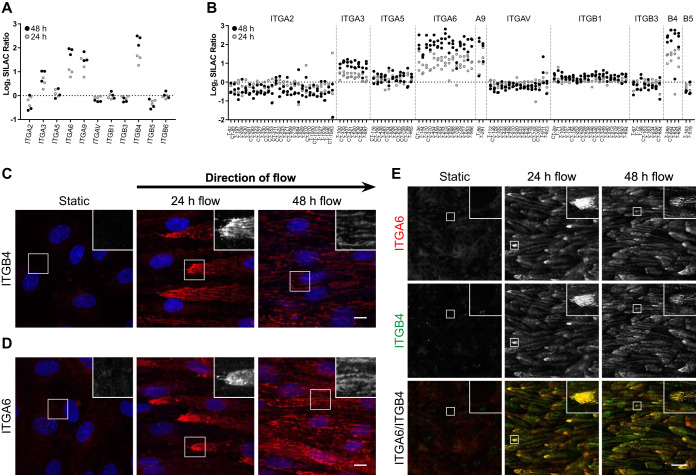
**Shear stress affects the distribution of matrix adhesion complex integrin α6β4.** *A*, Proteome and (*B*) cell surface proteome data of identified integrins. T or CT represents the enzyme used for protein digestion. The depicted numbers represent the biotin-modified lysine residue. *C*, *D*, Immunofluorescence maximum intensity projections of flow-treated (24 and 48 h) and statically-incubated ECs stained for integrin α6 and β4 (red). Blue is HOECHST. Scale bars are 10 μm. Inlays are twice enlarged. *E*, Co-localization of integrin α6 (red) and β4 (green). Depicted are maximum intensity projections. Micrographs are representative for 3 independent experiments. Scale bar is 50 μm. Inlays are 4× enlarged.

The composition of the ECM can drive the molecular make-up of the associated cell-matrix adhesion complexes, including the adhesion receptors (52). Most cell-matrix interactions are mediated by members of the integrin protein family (53), of which 11 subunits were identified by our MS approach. Three of these subunits, α6, α9, and β4, were significantly increased in flow conditions (Fig. 4*A*). These findings were confirmed in the cell surface proteome analysis (Fig. 4*B*), and indicate that the cell-matrix adhesion is affected by flow exposure. Next, we assessed the abundance and localization of the 2 integrin chains that were most prominently affected: α6 and β4. As expected, a clear increase in the fluorescent signal was observed for both integrin chains (Fig. 4*C*–4*D*), thereby confirming our MS data. Surprisingly, both integrin α6 and β4 showed an intense staining on the leading edge of the cells after 24 h of flow-exposure (Fig. 4*C*–4*D*). After 48 h of flow-exposure, a redistribution of these integrins had occurred into a mostly fibrillar pattern throughout the cells. Integrin α6 displayed a high degree of co-localization with integrin β4 (Fig. 4*E*), but not with β1 (supplemental Fig. S5).

**Fig. 5.**
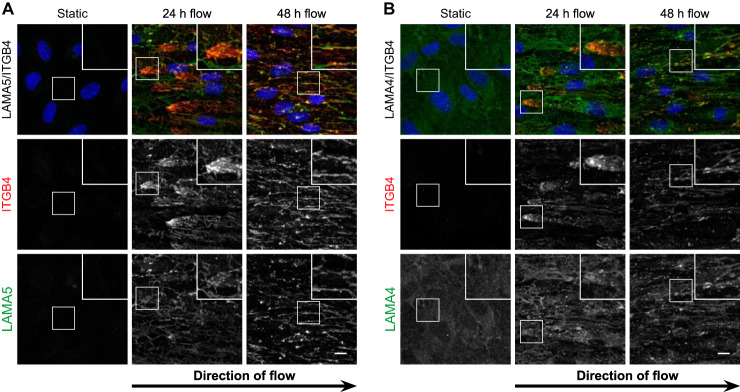
**Integrin α6β4 co-localizes with the laminin ECM.** Co-stainings of ITGB4 (red) and LAMA4 or LAMA5 (green). Blue is HOECHST. Depicted are maximum intensity projections. Micrographs are representative for 3 independent experiments. Scale bars are 10 μm.

Because integrin α6β4 has been shown to interact with LAMA5 (54), we next assessed the relative localization of these molecules. In line with the described interaction of integrin α6β4 and LAMA5, substantial co-localization of integrin β4 and LAMA5 was observed, particularly after 48 h of flow exposure (Fig. 5*A* and supplemental Fig. S6). Surprisingly, a similar co-localization was observed between this integrin heterodimer and LAMA4 (Fig. 5*B* and supplemental Fig. S7). A similar flow-induced localization of these proteins was observed in human lung microvascular cells and HUVECs (supplemental Fig. S8).

Taken together, these data indicate that the flow-induced remodeling of the endothelial BM is not limited to laminins, but includes altered protein levels and relocalization of laminin-associated proteins such as fibulins and integrins, and suggests the formation of a cell-matrix interaction complex via integrin α6β4 and LAMA5.

##### Flow-responsive Transcriptome Substantially Overlaps with Protein Signature

To assess whether the differences in protein levels correlated with altered RNA levels, we compared our results with recent transcriptomics data of flow-exposed endothelial cells. These data describe human umbilical vein endothelial cells (HUVECs) exposed to pulsatile shear (PS) or oscillatory shear (OS) flow in a parallel plate flow chamber. Of the flow-responsive proteins identified in our proteomics data, corresponding transcripts of 100 proteins were identified in this data set. Of these, 53 were affected by flow exposure both on protein and RNA level (supplemental Fig. S8*A*). Surprisingly, flow-induced changes in the levels of 490 other transcripts were not reflected in our data set. However, 442 of these were not different between pulsatile and oscillatory flow conditions, indicating that these changes are not specific for the flow type (supplemental Fig. S8*A*). To better understand the similarities and discrepancies between our proteomic data and the transcriptomics data set, we visualized the proteins that were affected in our experiments and the corresponding transcripts in a heatmap (supplemental Fig. S8*B*). This heatmap indicates that our proteomic data show overlap with transcriptomic data of endothelial cells cultured under pulsatile flow but not oscillatory flow conditions. For instance, the transcript levels of ITGB4 gradually increase in pulsatile flow conditions, but not oscillatory flow conditions (supplemental Fig. S8*C*). However, for other proteins, including ITGA6 and ITGA5, there is a discrepancy between the protein and RNA data (supplemental Fig. S8*C*). These may be the result of other regulatory mechanisms, such as the dimerization of ITGA6 and ITGB4.

##### Inflammation and Static Conditions Trigger Separate Pathways in ECs

**Fig. 6.**
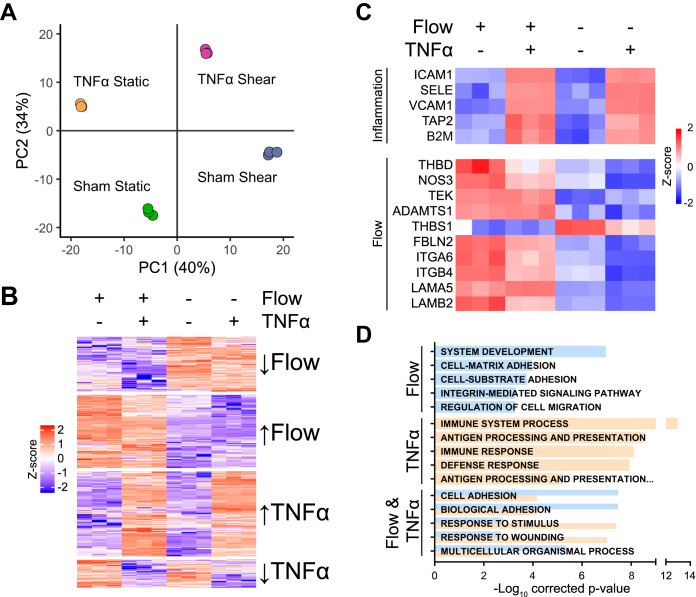
**Flow induces a specific protein signature that is unrelated to endothelial inflammation.** *A*, Principal component analysis of four groups of samples: static, TNFα-, flow-, and flow &TNFα-treated ECs. *B*, Heatmap representation of proteins changed significantly in either of the groups. Z-scored label-free quantification (LFQ) intensities are depicted in which red and blue represent increased and decreased values, respectively. *C*, Heatmap representation of z-scored LFQ values of hallmark proteins of TNFα and flow-exposure. *D*, Top 5 unique and shared GOterms enriched in affected proteins of TNFα- or flow-treated samples. A pool of BOECs originating from 3 healthy volunteers was used. Blue GO-terms are enriched in flow conditions, salmon upon TNFα treatment.

We previously showed that TNFα stimulation of ECs affects ECM-related proteins and integrin subunits ITGA6 and ITGB4 as well (27). These results are of particular interest as vascular inflammation and flow-exposure may be interlinked, because an inflammatory signature has been detected in regions of the vascular bed exposed to disturbed flow or low shear stress (14, 55). To dissect the interplay between a lack of flow and inflammation at a system-wide level, we compared the proteomes of ECs exposed to flow to ECs in static conditions, ECs exposed to TNFα, and ECs in static conditions exposed to TNFα, by employing label-free proteomics. Using this approach, we quantified the abundance of 5613 proteins, of which 533 changed among the different conditions (supplemental Table S5). A highly dissimilar effect was observed for a lack of flow and TNFα stimulation compared with unidirectional flow-exposed cells in a principal component analysis (Fig. 6*A*) and k-means clustering of differentially abundant proteins (Fig. 6*B*). Hallmark inflammatory proteins, such as ICAM1 (intercellular adhesion molecule 1), VCAM1 (vascular cell adhesion molecule 1), and SELE (E-selectin), were specifically increased upon exposure to TNFα (Fig. 6*C*, top panel), whereas established flow-associated proteins, including NOS3, THBD and TEK, as well as the flow-responsive proteins identified in this study, were specifically in- or decreased in unidirectional flow conditions (Fig. 6*C*, bottom panel). As expected, affected proteins were enriched for cell-matrix adhesion in flow-exposed ECs and for immune-related processes in TNFα-stimulated ECs, respectively (Fig. 6*D*, supplemental Table S6). To focus on the inflammatory signature that was observed in regions of disturbed flow *in vivo*, we selected proteins that were similarly affected by a lack of flow and inflammation. In total, 32 proteins were affected in a similar manner by a lack of flow and TNFα, including ITGB4 and FBLN2 (supplemental Fig. S9). Combined, these results indicate that a lack of flow and TNFα-exposure exert fundamentally different effects on ECs.

## DISCUSSION

Hemodynamic forces are crucial for the function of the endothelium in vascular homeostasis and thereby for vascular health. Despite this importance, limited information is available on the flow-responsive molecular wiring in vascular endothelial cells. Here, we present quantitative proteomic data of ECs exposed to flow for 24 and 48 h. We employed Blood Outgrowth Endothelial Cells for their superior propagation capacity (26), which was necessary to obtain >95% SILAC amino acid incorporation required for protein quantification. Our MS data confirmed the flow-induced regulation of nitric oxide production and cytoskeletal rearrangements. In addition, we now show that flow exposure results in extensive remodeling of the endothelial basement membrane, the formation of a laminin network, and expression of the ECM-adhesion complex integrin α6β4. Furthermore, our data indicate that, *in vitro*, lack of flow does not induce an inflammatory signature. This study provides an extensive resource on the hemodynamic response of endothelial cells and emphasizes the role of the subendothelial basement membrane in flow-adaptation.

In line with previous studies that have demonstrated a crucial role for LAMA5 in EC alignment and production of vasodilatory agents in response to flow (56), our data indicate that the expression of LAMA5 is increased in flow conditions. This laminin has been shown to decrease the transmigration capacity of leukocytes both directly, by inhibiting the migration across the BM (57), and indirectly, by improving the barrier function of the associated ECs (58). This suggests that leukocytes will primarily transmigrate in regions devoid of high shear stress. In line with this hypothesis, leukocyte extravasation mainly takes place in post-capillary venules, which are not exposed to high shear forces (59) and contain low levels of LAMA5 (60). It is therefore tempting to speculate that the low LAMA5 content of these vessels is the direct result of low shear force exposure.

In this study, our combined proteomic and immunofluorescence data reveal that, in addition to the increased LAMA5 levels, the protein expression levels, and localization of multiple other BM-related proteins are affected in flow conditions. Notably, both LAMA4 and LAMA5 adopted a network-like phenotype in flow conditions. A possible explanation for this observation lies in the N-terminal region of LAMA5, which has the capacity to bind the N-terminal regions of laminin β- and γ-chains, thereby supporting the polymerization of LAMA5-containing heterotrimers (61) into a laminin network. Remarkably, although LAMA4 lacks such a polymerization region (48, 62, 63), it also adopted a network configuration, however, exclusively when ECs were exposed to flow. We speculate that the expression of LAMA5 induces the formation of a laminin network, which also induces the relocalization LAMA4, possibly via the N-terminal polymerization regions of the β- and γ-subunits of the LAMA4-heterodimers.

In addition to the endothelial BM, our MS data indicated that various proteins involved in matrix adhesion were affected in flow conditions. Within the integrin family we detected a flow-induced increase in the expression levels of α3, α6, α9, and β4 subunits, of which α3, α6, and β4 form specific receptors for laminins (64). This may suggest that in flow conditions, besides binding to collagen and the RGD-motif, ECs rely strongly on laminin-binding integrins for cell adhesion. This agrees with the recent finding that the simultaneous blockage of integrin α3 and α6 results in an abrogation of the EC adhesion capacity to laminin 511 (56). As the α6 and β4 subunits were most affected in our MS data, we subsequently focused on these, and showed that integrin α6β4 and LAMA5 co-localized in flow conditions. Combined with the finding that purified integrin α6β4 and LAMA5 interact (54), this suggests that these proteins may be part of the same complex, and that integrin α6β4 plays a role in cell adhesion to LAMA5 (65). Integrin dimers are involved in various adhesion structures, including focal complexes, focal adhesions (66), fibrillar adhesions (67, 68) and the recently described reticular adhesions (69). These structures can be dynamic in their integrin content, however, the specific flow-induced integrin α6β4 pattern did not match any of these structures. A possible explanation for this unknown integrin localization may be found in the laminin network formation, as integrins and their laminin substrates have previously been shown to affect each other's localization in a reciprocal fashion (70). The question remains what the function of this complex is. Integrin α6β4 is well-known in epithelial cells for its role in hemidesmosomes: multiprotein complexes that link intracellular keratin intermediate filaments to the ECM laminin 332 (71). These complexes are essential in maintaining epithelial adhesion to the BM, and their disruption results in the blistering disease junctional epidermolysis bullosa and other severe problems involving epithelia (72, 73). Would integrin α6β4 serve a similar purpose in ECs? It has been described that integrin α6β4 is expressed by ECs *in vivo* (74), and that overexpression of this integrin results in the formation of adhesion complexes that link to the laminin BM and the vimentin cytoskeleton via plectin (75, 76). Furthermore, it has been shown that laminin 511 supported cell adhesion in flow conditions, whereas laminin 411 did not (56). As integrin α6β4 can interact with laminin 511, but not 411 (54), these data may suggest that, in endothelial cells, the LAMA5-integrin α6β4 complex anchors ECs to the BM. This is supported by the observation that plectin (PLEC), which forms a link between integrin β4 and the vimentin cytoskeleton (76), displays a higher abundance in flow-exposed cells. We therefore speculate that the LAMA5-integrin α6β4 link enables the cells to withstand higher shear forces.

In addition to ECM- and adhesion-related proteins, various other proteins were affected by flow-treatment, for which the function and molecular interactions are yet to be determined. For instance, parallel to the proteolytic processing of LAMA4, protein levels of metalloprotease MMP15 were increased. Other membrane-type MMPs have been shown to cleave various ECM components (77) including laminin 111 (78). It is therefore tempting to speculate that MMP15 may have a role in LAMA4 processing. Furthermore, two proteins that are associated with the barrier function were affected: CLDN11 (claudin 11) and NDRG1 (N-Myc downstream regulated 1). Knock-down of NDRG1 has previously been associated with a decrease in epithelial barrier function (79), whereas CLDN11 is part of the tight junctions and thereby maintains the endothelial barrier function (80). Possibly, the altered protein levels we observed for CLDN11 and NDRG1 may therefore affect the endothelial barrier function in flow conditions.

In this study, we focused on the endothelial transition from a polygonal to an elongated and aligned phenotype using a ring-shaped culture dish (81). This morphological change requires planar polarization of ECs, yet it remains to be elucidated how this polarization is initiated. Multiple proteins and structures have been reported to be required for this process, including VE-cadherin and PECAM1 (82), polarity protein GPSM2 (also known as LGN) (83), microtubules (84) and integrin-complexes (85). Our observation that the integrin α6β4 adhesion complex is initially localized at the leading edge may provide a starting point for further studies into the flow-induced polarization of ECs.

Combined, our data show that, in addition to the morphological change, improved barrier function and regulation of the vasomotor tone, flow exposure of ECs results in a drastic remodeling of the endothelial BM and the cell-BM interaction. This change is characterized by LAMA5-driven laminin network formation and cell-matrix adhesion via integrin α6β4. These findings illustrate the plasticity of the endothelial BM and emphasize the role of blood flow in the vascular function. Additional studies are required to further dissect the implications of the observed integrin-laminin co-localization, including interventional studies using depletions of ITGA6, ITGB4, and LAMA5 to unravel their role in EC functions such as NO production, cell alignment and leukocyte transmigration. In addition, it is essential to assess the EC proteome in other flow conditions, including oscillatory, laminar, pulsatile and turbulent flow, and under different flow rates. Moreover, it will be important to evaluate whether these data can be extrapolated to ECs from distinct vascular beds, including aortic ECs and ECs originating from post-capillary venules. Finally, studies using EC-specific knock-out mice are required to answer the question how our findings translate to *in vivo* settings.

An important aspect of mechanosensing in a (patho)physiological context is the interplay between flow and inflammation. *In vivo*, regions of the vasculature not exposed to laminar flow have been shown to display a pro-inflammatory signature, including increased expression of adhesion proteins ICAM1 and VCAM1 (86–88). Indeed, multiple *ex vivo* and *in vitro* studies suggest that flow can affect aspects of the endothelial inflammatory response (5–10). Our data now show that, at a system-wide level *in vitro*, flow and inflammation trigger a fundamentally distinct endothelial response showing limited interplay. Although we cannot exclude that the apparent discrepancies between our results and the aforementioned studies may result from differences in experimental design, our data suggest that the absence of flow does not result in an inflammatory signature within our experimental timeframe. Possibly, other factors in the complex pathophysiological environment may contribute to the development of diseases related to flow deprivation (89). Further research into the endothelium in its (patho)physiological environment, possibly using ECs harvested from patients with vascular diseases, is therefore needed to fully understand the basis of vascular disorders such as atherosclerosis and thrombosis.

## DATA AVAILABILITY

The raw MS files and search/identification files obtained with MaxQuant have been deposited in the ProteomeXchange (90) consortium via the PRIDE partner repository (91) (https://www.ebi.ac.uk/pride/archive/) with identifier PXD014526.

## Supplementary Material

Table S1 Flow-induced proteomic alterations in ECs.

Table S2 Flow-induced changes in the cell surface proteome, unambiguous localizations.

Table S3. Flow-induced changes in the cell surface proteome, ambiguous localizations.

Table S4. GO-term enrichment analysis of proteins affected by flow.

Table S5. Flow- and TNFa-induced proteomic alterations in ECs.

Table S6. GO-term enrichment analyses of flow- and TNFα-induced changes in the EC proteome.

Table S7-S14

Supplemental data
