# Supplementary material for: Flow-induced Reorganization of Laminin-integrin Networks Within the Endothelial Basement Membrane Uncovered by Proteomics
Source: Mol Cell Proteomics. 2020 Apr 24;19(7):1179–92. doi: 10.1074/mcp.RA120.001964 (PMC7338090; doi:10.1074/mcp.RA120.001964)
Supplement: Supplemental data [file 158553_1_supp_511757_q87020.pdf]

Flow-induced reorganization of laminin-integrin networks within the endothelial basement membrane uncovered by proteomics

Eelke P. Béguin, Esmée F.J. Janssen, Mark Hoogenboezem, Alexander B. Meijer, Arie J. Hoogendijk, Maartje van den Biggelaar

Supplemental data

Supplemental figures

Figure S1. The cell surface biotin label is mainly present at the apical membrane of ECs.
Figure S2. The cell surface proteome corresponds to the proteome and enriches for cell surface proteins.
Figure S3. LAMA4 and LAMA5 co-localize in flow conditions.
Figure S4. Integrin β1 distribution does not correspond with integrin α6 localization.
Figure S5. Tile scan projections of ITGB4 and LAMA5 localization in flow-exposed BOECs.
Figure S6. Tile scan projections of ITGB4 and LAMA4 localization in flow-exposed BOECs.
Figure S7. Flow induces ITGB4 expression and initial leading edge localization in HMVECs and HUVECs.
Figure S8. Flow-responsive transcriptome substantially overlaps with protein signature.
Figure S9. Proteins displaying opposing and synergistic effects upon flow-deprivation and TNFα-stimulation.

Table S1. Flow-induced proteomic alterations in ECs.
Table S2. Flow-induced changes in the cell surface proteome, unambiguous localizations.
Table S3. Flow-induced changes in the cell surface proteome, ambiguous localizations.
Table S4. GO-term enrichment analysis of proteins affected by flow.
Table S5. Flow- and TNFa-induced proteomic alterations in ECs.
Table S6. GO-term enrichment analyses of flow- and TNFα-induced changes in the EC proteome.
Table S7. All assigned peptides from the SILAC proteome data (Table S1).
Table S8. All assigned proteins from the SILAC proteome data (Table S1).
Table S9. All assigned peptides from the trypsin-digested biotin pull-down (Table S2-3).
Table S10. All assigned proteins from the trypsin-digested biotin pull-down (Table S2-3).
Table S11. All assigned peptides from the chymotrypsin-digested biotin pull-down (Table S2-3).
Table S12. All assigned proteins from the chymotrypsin-digested biotin pull-down (Table S2-3).
Table S13. All assigned peptides from the label-free protein quantification of cells stimulated with flow, TNFα or both (Table S5).
Table S14. All assigned proteins from the label-free protein quantification of cells stimulated with flow, TNFα or both (Table S5).

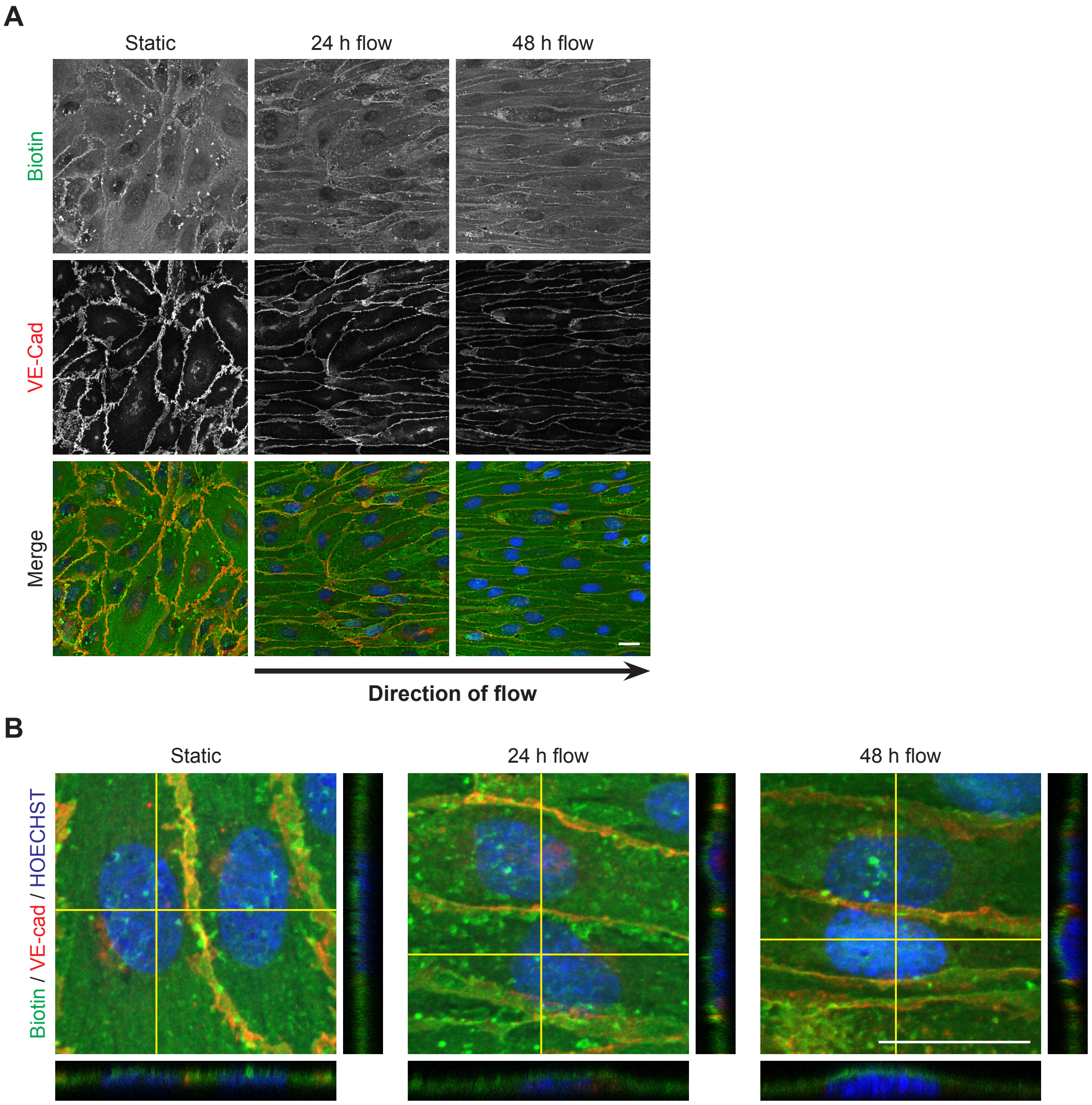

Figure S1. The cell surface biotin label is mainly present at the apical membrane of ECs. (A) Immunofluorescence maximum intensity projections of BOECs subjected to flow for 0, 24 or 48 hours and labeled with sulfo-NHS-LS-biotin. The scale bar is 20 μm. Cells were stained for biotin (green), VE-cadherin (red) and with HOECHST (blue). (B) Staining as in (A), now depicted with an orthogonal projection on the location of the yellow lines. The scale is 20 μm.

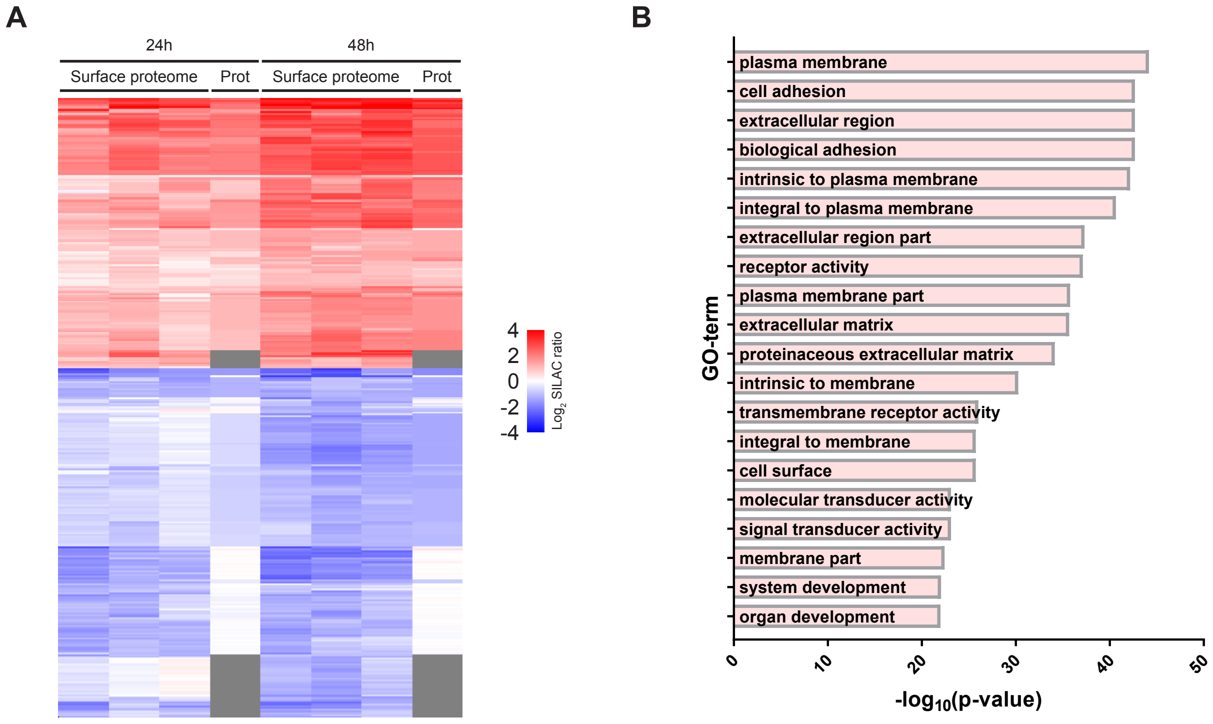

Figure S2. The cell surface proteome corresponds to the proteome and enriches for cell surface proteins.
(A) Heatmap representation of biotin sites that were significantly affected in flow conditions. Each row represents a biotinylates site. Biotin sites have been matches to the corresponding median proteome SILAC ratios. (B) Top 20 enriched GO-terms in the cell surface proteome compared to the whole cell proteome and surface proteome combined.

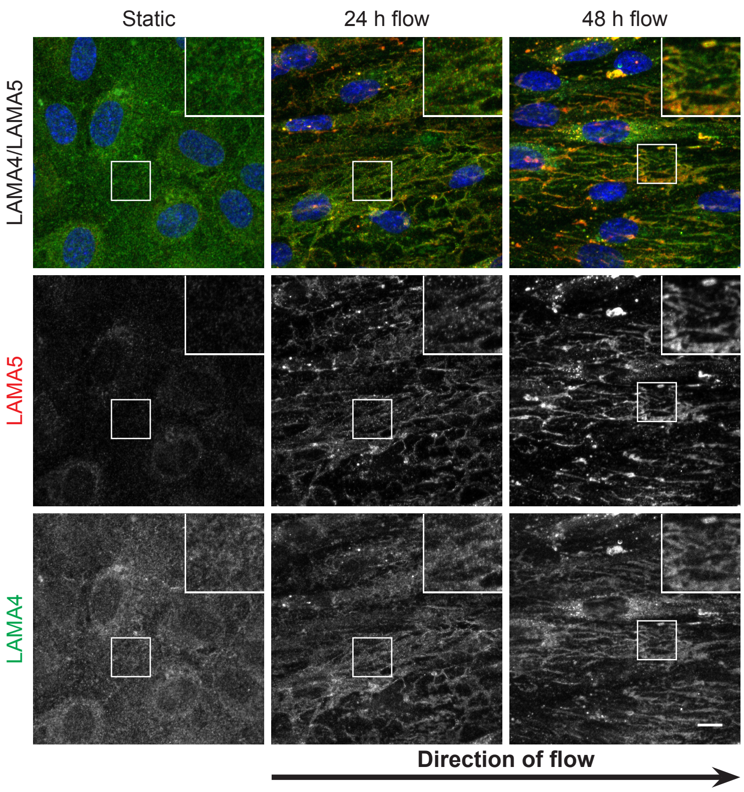

Figure S3. LAMA4 and LAMA5 co-localize in flow conditions.
Co-stainings of LAMA4 (green) and LAMA5 (red). Blue is HOECHST. Depicted are maximum intensity projections, scale bars are 10 μm.

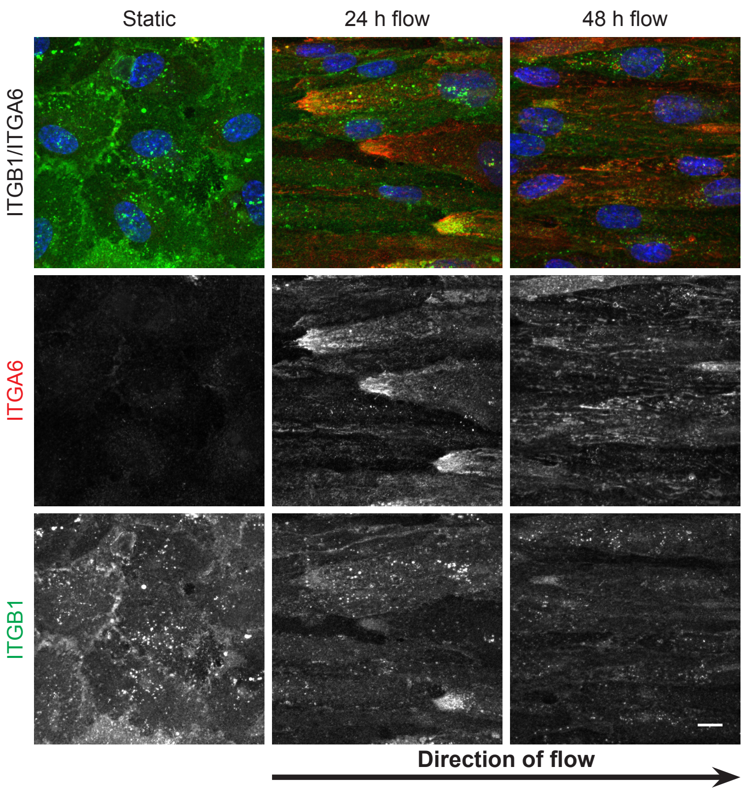

Figure S4. Integrin β1 distribution does not correspond with integrin α6 localization. Co-staining of ITGA6 (red) with ITGB1 (green). Blue is HOECHST. Depicted are maximum intensity projections. The scale bar is 10 μm.

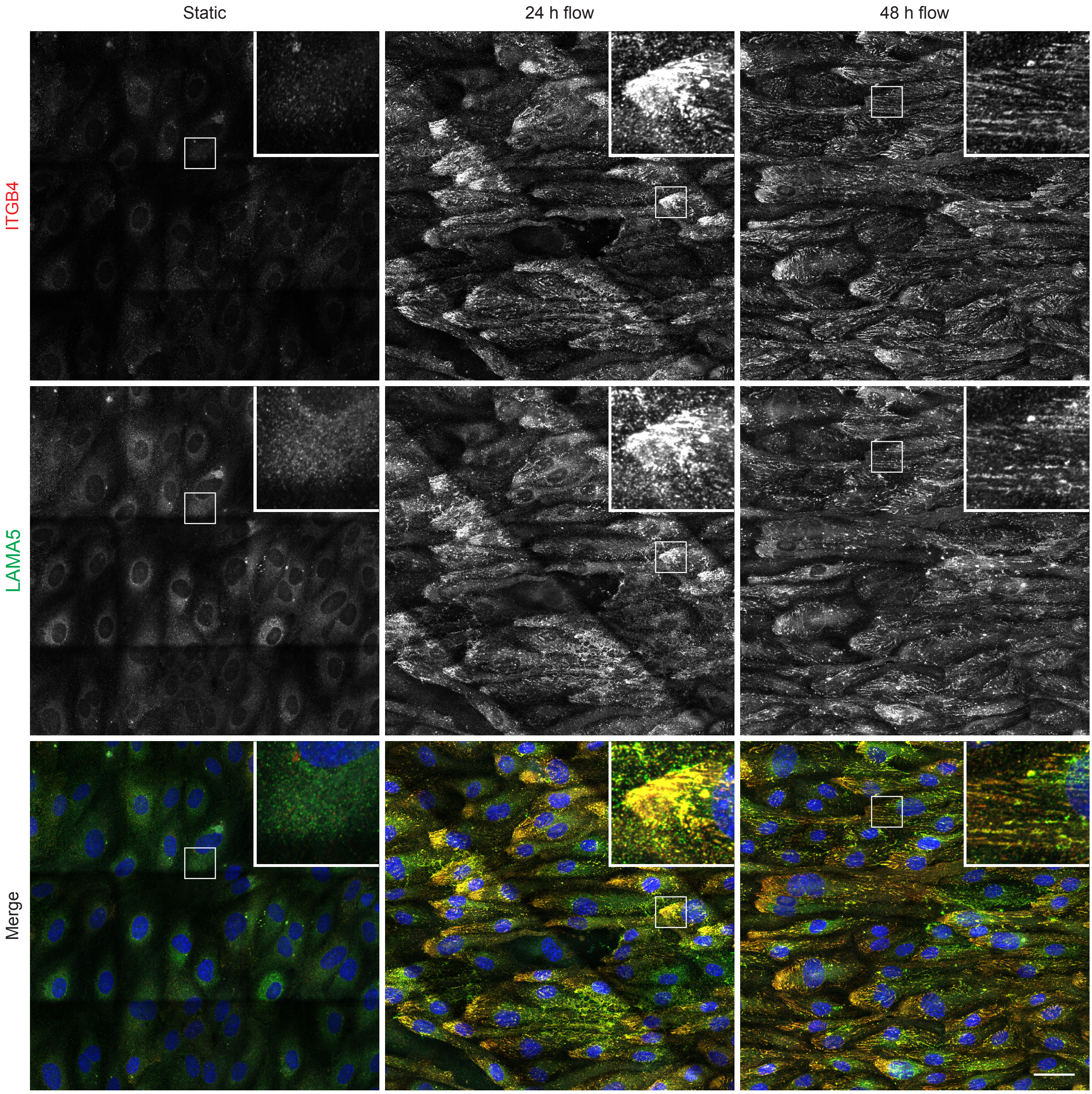

Figure S5. Tile scan projections of ITGB4 and LAMA5 localization in flow-exposed BOECs. Co-staining of ITGB4 (red) and LAMA5 (green). Blue is HOECHST. Depicted are maximum intensity projections of tile scans. The scale bars are 20 µm.

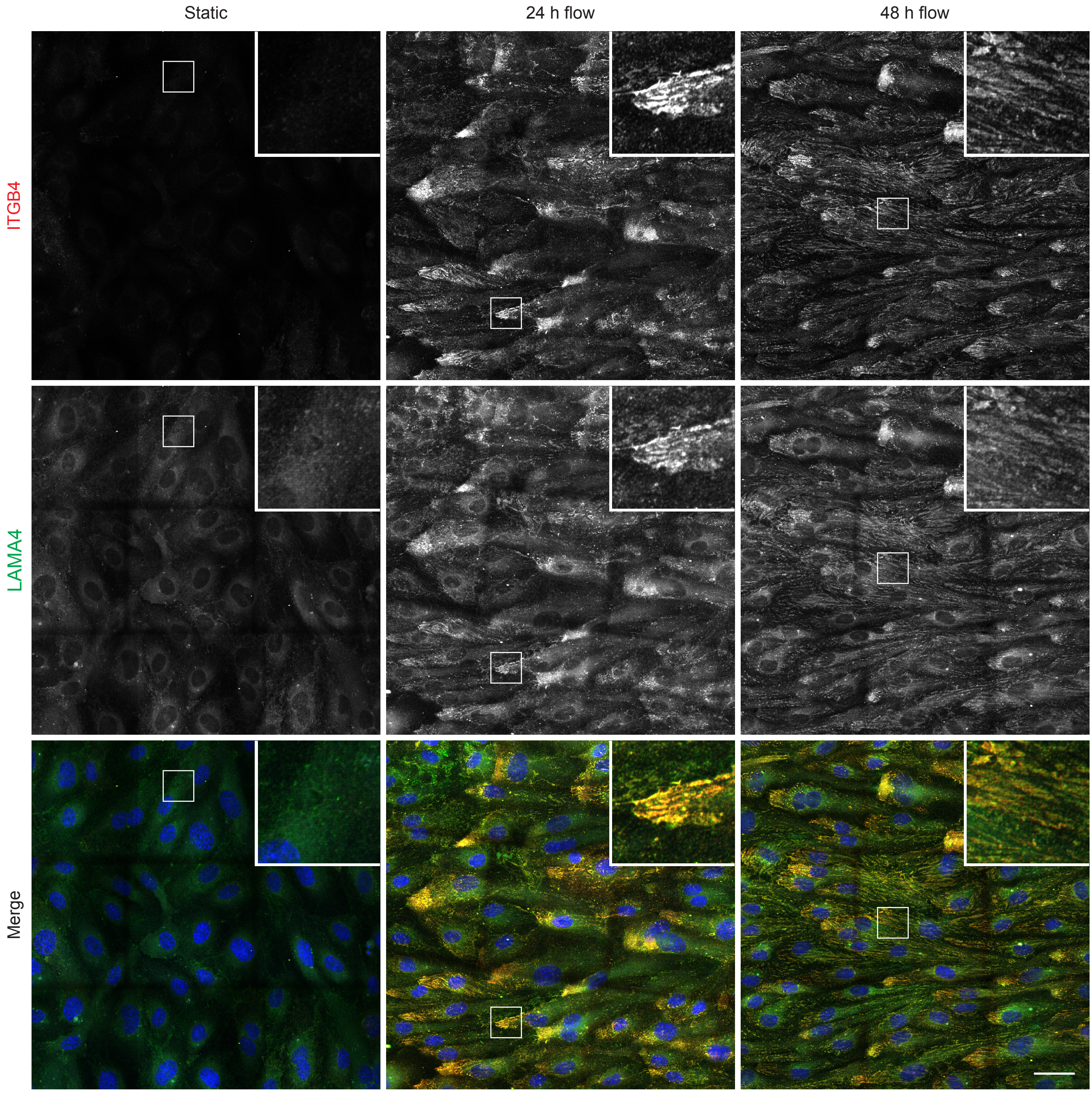

Figure S6. Tile scan projections of ITGB4 and LAMA4 localization in flow-exposed BOECs. Co-staining of ITGB4 (red) and LAMA4 (green). Blue is HOECHST. Depicted are maximum intensity projections of tile scans. The scale bars are 20 µm.

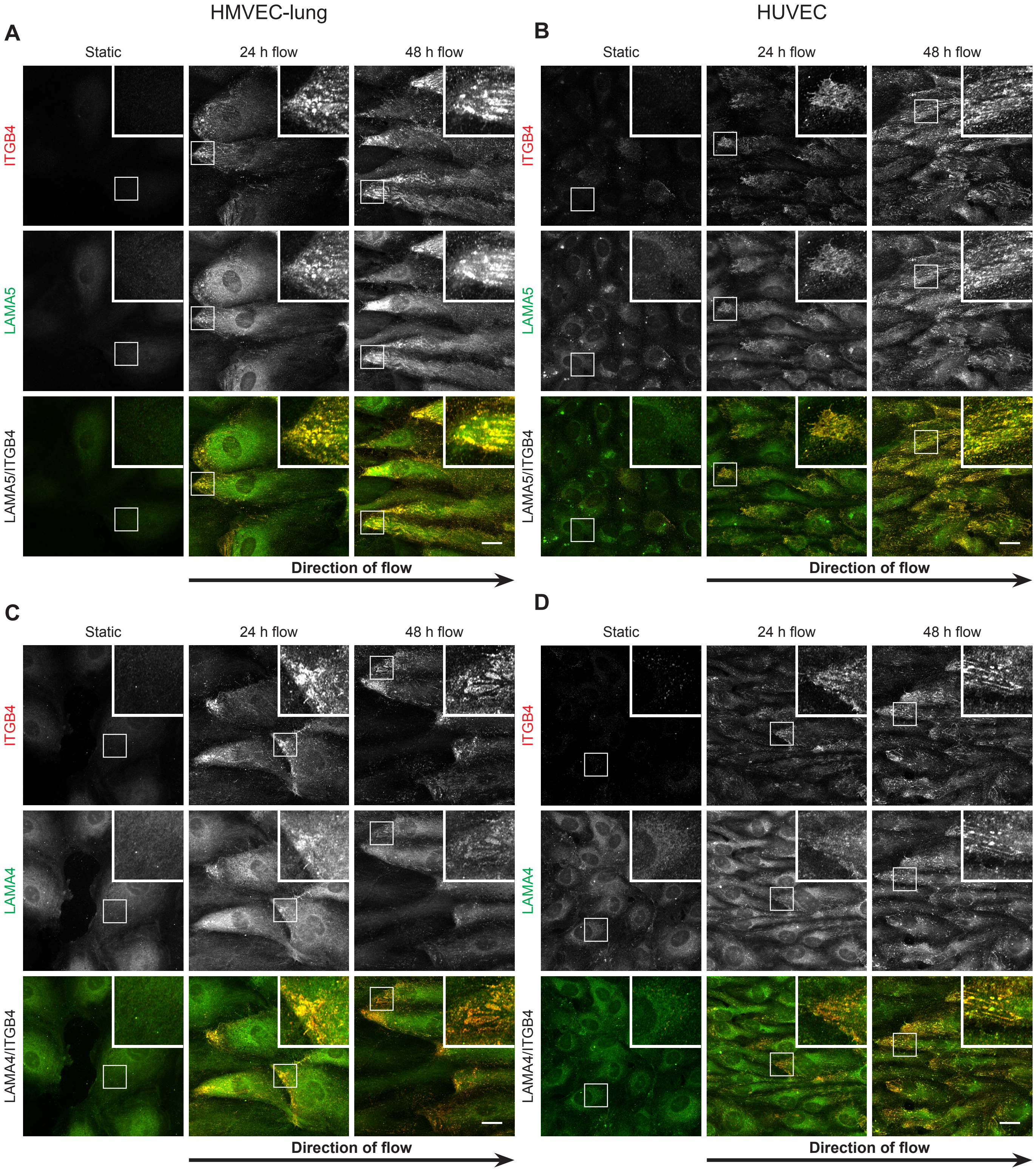

Figure S7. Flow induces ITGB4 expression and initial leading edge localization in HMVECs and HUVECs.

Co-stainings of LAMA4 (panel A-B) or LAMA5 (panel C-D, green) and ITGB4 (red). Depicted are maximum intensity projections, scale bars are 20 µm.

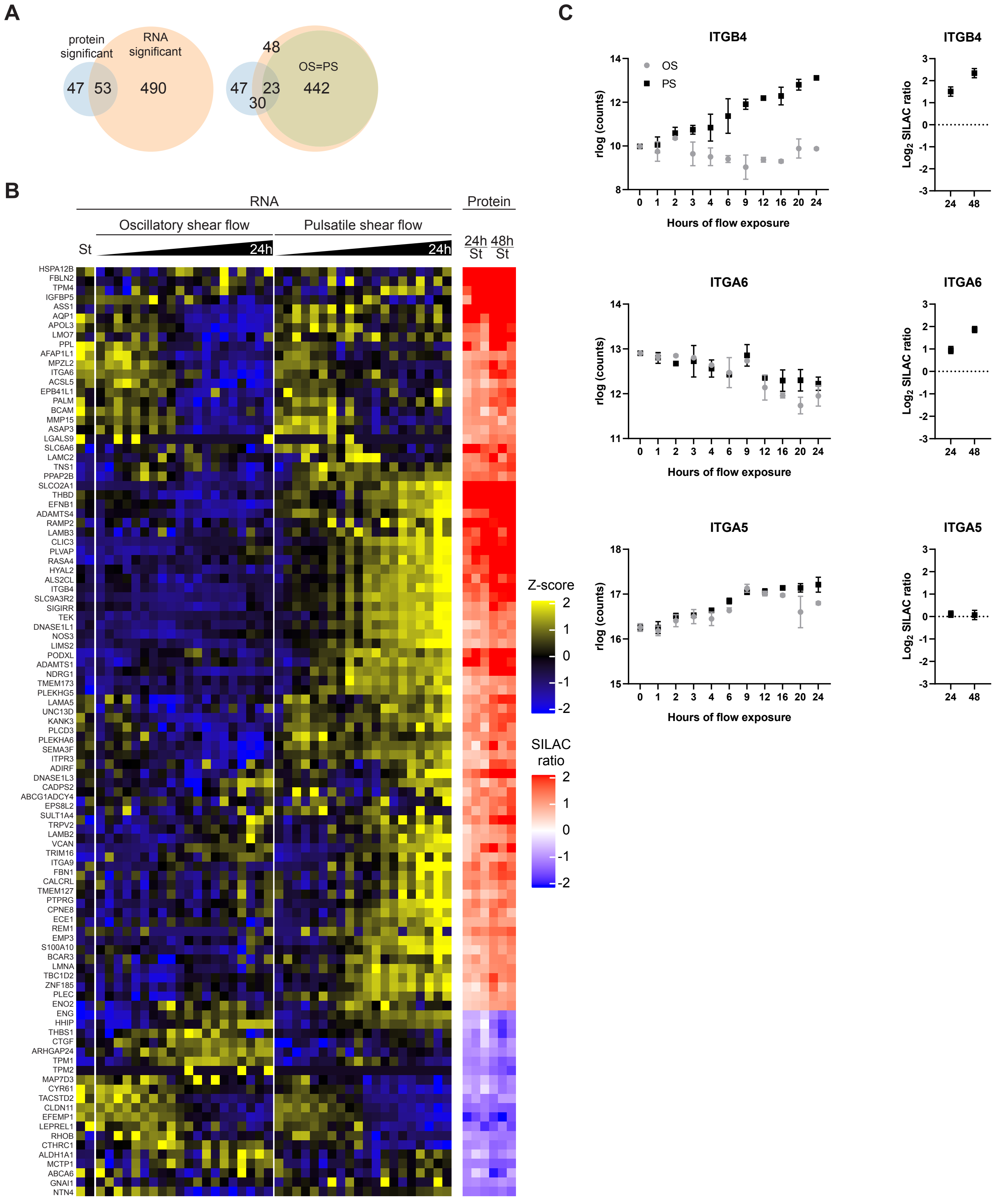

Figure S8. Flow-responsive transcriptome substantially overlaps with protein signature. Comparison of the proteomics data presented in this study with transcriptomics data of flow-exposed HUVECs of Ajami *et al.* (25). (A) Comparison of significant proteins/transcripts identified in both studies. OS = oscillatory shear flow, PS = pulsatile shear flow. OS=PS population did not display a significant difference between the two flow conditions. (B) Heatmap representation of flow-responsive proteins of which transcriptomics data was available as well. (C) Transcriptomics and proteomics data of selected proteins.

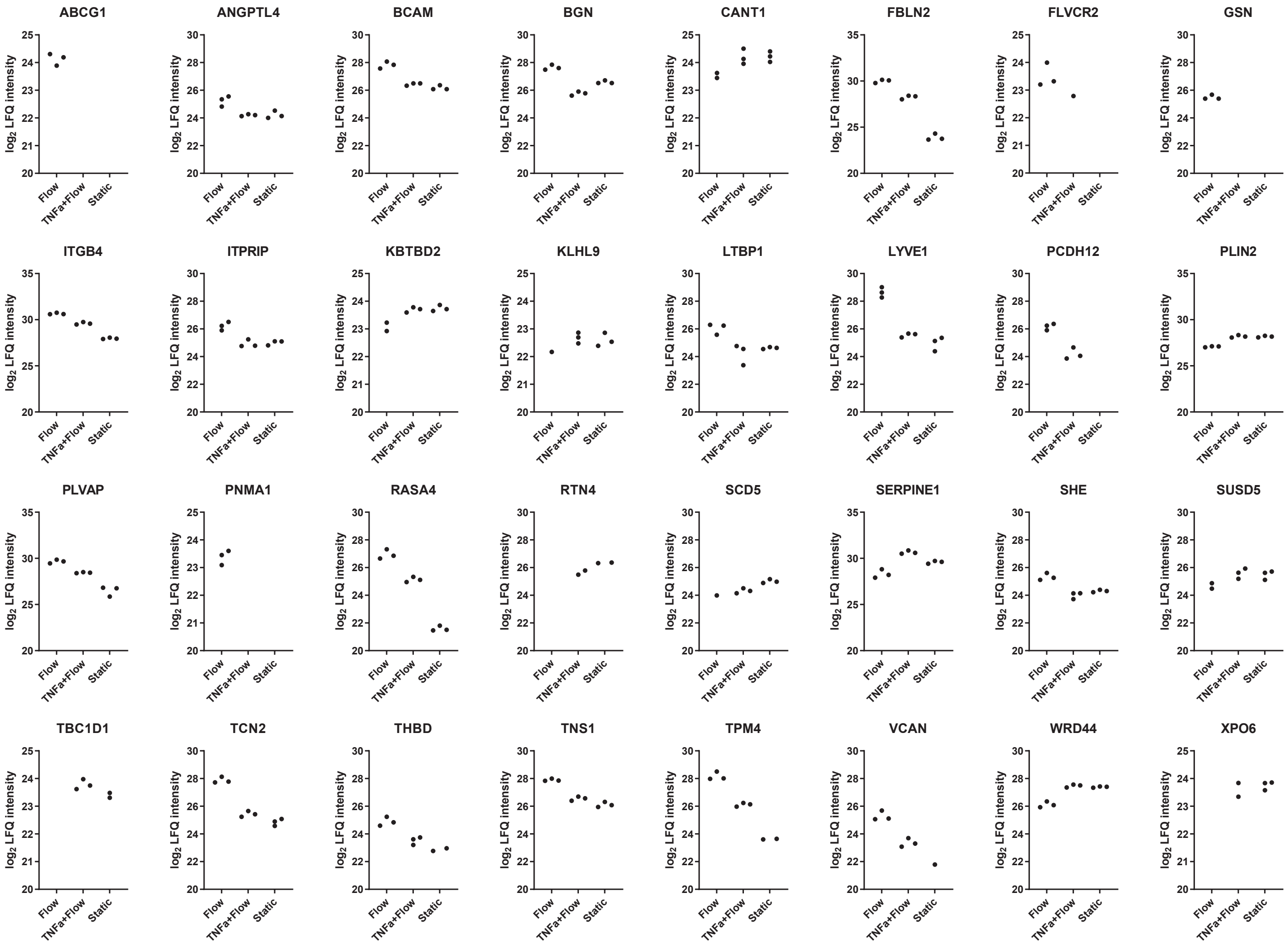

Figure S9. Proteins displaying similar effects in static conditions and TNFα-stimulation. Log_2 LFQ intensities of proteins that were significantly different compared to flow conditions upon static incubation or TNFα exposure.
